# The Port Alfred floods of 17–23 October 2012: A case of disaster (mis)management?

**DOI:** 10.4102/jamba.v8i1.207

**Published:** 2016-04-26

**Authors:** Desmond M. Pyle, Tennielle L. Jacobs

**Affiliations:** 1 School of Disaster Management, Stenden University, South Africa; 2 Port Elizabeth Weather Office, South African Weather Service, South Africa

## Abstract

An intense cut-off low weather system, more commonly known regionally as a ‘black south-easter’, caused severe flooding in Port Alfred and the surrounding coastal areas from 17 to 23 October 2012. Unconfirmed reports of up to 700 mm of rainfall for the period were recorded. Damage caused by the flooding was estimated at R500 million. Eight deaths were recorded. The poorly maintained and ageing infrastructure and storm water systems could not withstand the floodwaters, and as a result, damage was worse than it should have been. Many houses, particularly in the surrounding townships and informal settlements, were destroyed. Disease threats arose, including cholera, diarrhoea and influenza. The South African Weather Service issued weather warnings of severe local flooding in the coastal areas of the Eastern Cape a few days before the flood event. Unfortunately, there was a delay in communicating the severe weather warning effectively to the public, relevant authorities and role-players by local disaster management officials. In addition, there was poor and ineffective local coordination of disaster response and relief efforts. This paper examines the 2012 flood event from both meteorological and disaster management perspectives, using a combined qualitative and quantitative research approach. Findings point to a critical lack of coordination amongst the various role-players before, during and after the disaster. Recommendations for improved proactive and coordinated disaster risk management and disaster risk reduction for the region are made.

## Introduction

**FIGURE 1:**
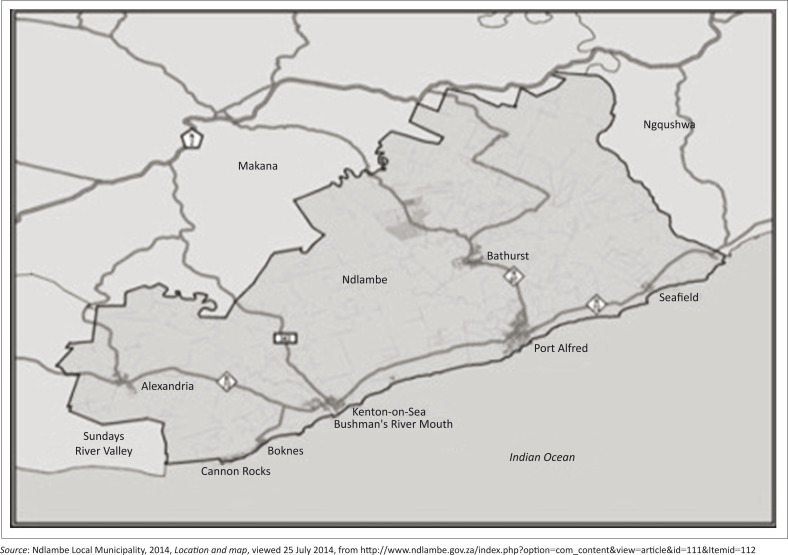
The location of Port Alfred within Ndlambe Municipality.

Port Alfred is situated on the coast at the mouth of the Kowie River, in the Ndlambe Municipal District in the Eastern Cape Province of South Africa (Figure 1). It is primarily a holiday town, with a number of permanent retirees and limited commercial or industrial development. Its total population is approximately 26 000. A large proportion of the population lives in the surrounding townships, which are generally poor, and unemployment is common. The town itself has a wealthier population, of which the most affluent reside in the upmarket residential marina development along the Kowie River, close to the river mouth. Port Alfred and the surrounding area exhibit great inequalities in wealth and vulnerability to weather-related hazards, which is reflective of the broader poverty in the Eastern Cape Province (Pyle 2006).

Port Alfred and the surrounding coastal areas have been regularly affected by flood events in the past. The majority of these events are caused by extreme cut-off low weather systems (Singleton & Reason 2006; Taljaard 1985), which can cause major infrastructural damage and widespread disruption to the coastal towns (Molekwa 2013).

Disaster management structures exist at local level (Ndlambe Municipality), under guidance from district level (Cacadu Municipality). The local municipality is responsible for the coordination of disaster management and disaster risk reduction measures for Port Alfred and the broader Ndlambe region. It is severely under-resourced and under-staffed (it has only one full-time official), and as a result responsibility for coordination often needs to be taken at district level (D. Shuping, [Provincial Disaster Management Centre: Eastern Cape], pers. comm., 13 July 2014). Consequently, when flood events occur in the region, local coordination and relief efforts by Ndlambe Municipality are often weak and ineffective. In such cases, relief efforts are sporadic and *ad hoc*, and other actors such as the police, the military, traffic police, local businesses and volunteer or civic organisations, such as the National Sea Rescue Institute (NSRI), have to take responsibility for managing relief and recovery efforts.

According to the Ndlambe Municipality’s Final IDP 2012–2017 (Ndlambe Local Municipality [NLM] n.d.), a number of factors increase the vulnerability of the municipality’s residents to flooding:

- the location of settlements on floodplains, slopes and low-lying areas
- the high number of households living in informal settlements
- the lack of awareness of flood hazards
- the lack of maintenance and cleaning of storm water systems or insufficient capacity of the systems
- no early warning systems.

## Problem statement and research objectives

The Port Alfred floods of October 2012 serve as an example of the problems and difficulties relating to effective and coordinated disaster relief efforts in the aftermath of severe flooding in the region.

In light of the context provided in the previous section, the following two research objectives have been formulated:

- to analyse the meteorological conditions which had disastrous impacts on deeply unprepared and poorly settled populations
- to review critically the functions and contributions of the various role-players during the flood event, in particular disaster management structures, municipal officials and other actors and agencies.

### Literature review

#### Disaster management and disaster risk reduction at a local level

The *National Disaster Management Act* (No 57 of 2002) (Republic of South Africa [RSA] 2002), the National Disaster Management Framework (RSA 2005), and the Disaster Management Amendment Bill (RSA 2013) all emphasise the importance and the responsibility of local authorities in pro-actively managing disaster risk. In the event of local authorities not being able to cope with the magnitude of a severe event, assistance may be requested from district, provincial and finally national disaster management structures. It is important to note, however, that the responsibility for the coordination of mitigation and relief activities before and after disasters lies with the local disaster management authorities (RSA 2005). It is therefore vitally important that local disaster management offices are properly staffed and well resourced to deal proactively in implementing measures aimed at reducing risk to any common hazards experienced in the region.

#### Early warning of flood events

The South African Weather Service (SAWS) is the legal sole provider of severe weather warnings, according to the *South African Weather Service Act* (No 8 of 2001) (Poolman 2006) and the more recently promulgated South African Weather Service Amendment Bill (RSA 2011). In the event of impending severe weather, the SAWS issues weather advisories, watches and warnings to the relevant disaster management authorities and to the broader public (Poolman 2006). Severe weather warnings are disseminated by radio, SMS (short message service), television, other media and on the official SAWS website (Poolman n.d.).

There have been dramatic improvements in weather forecasting science in the last few decades, predominantly as a result of the use of numerical weather prediction (NWP) and ensemble prediction systems (Poolman n.d.). This has led to improved severe weather warnings and increased lead-times, which is critical for disaster management authorities in mobilising forces to respond to any impending weather disaster. Recently there has been a move to a multi-hazard and multi-sector approach towards severe weather early warnings (Poolman n.d.). This focuses on integrated collaboration between forecasters, disaster management structures and relief organisations, and understandable and useful warnings reaching the general public and affected communities in time.

#### Cut-off low events in South Africa, focusing on Port Alfred and the south-eastern coastal region

**FIGURE 2:**
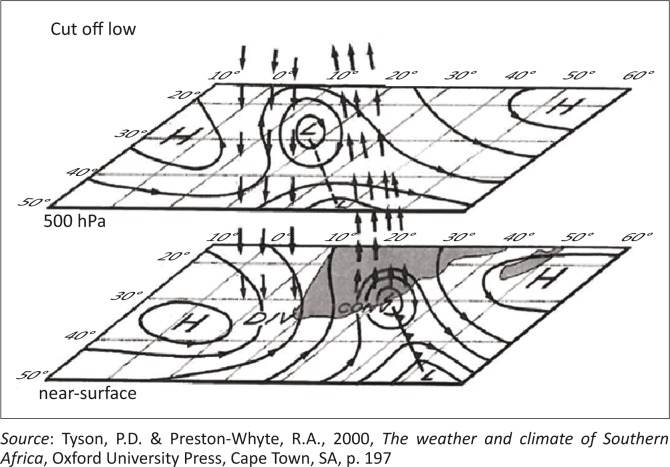
Cut-off low circulation patterns at near surface at 500 hPa levels.

Numerous authors (Molekwa 2013; Singleton & Reason 2006; Taljaard 1985; Tyson & Preston-Whyte 2000) highlight the significance of cut-off lows as rain-producing, and potentially flood-producing, systems in the south-eastern coastal region of South Africa. Whilst cut-off lows may be regarded as extreme weather-producing systems, they are not freak or unexpected in the region and are reasonably predictable over time and space (Nott 2006). Although many definitions of cut-off lows exist in the literature, the following description by Tyson and Preston-Whyte (2000) and accompanying figure (Figure 2) best outline their attributes and formation:

[*A cut-off low is a*] more intense form of westerly trough, a feature that is a cold-cored depression, which starts as a trough in the upper westerlies and deepens into a closed circulation extending downward to the surface and which becomes displaced equatorward out of the basic westerly current. Cut- off lows are unstable, baroclinic systems that slope to the west with increasing height and are associated with strong convergence and vertical motion, particularly while they are deepening. (pp. 196–197)

In simpler terms, cut-off lows result in cyclonic circulation which becomes detached from the predominant flow, often resulting in heavy rainfall, gale-force winds and other severe weather. Historically, the most severe cut-off low affecting the south-eastern coast occurred on 1 September 1968, when up to 560 mm of rainfall fell in 24 hours in Port Elizabeth, causing R40 million damage and 11 fatalities (SAWS 1991). Other notable flood events arising from cut-off lows were those in Laingsburg (1981), KwaZulu-Natal (1987), Montagu (2003) and the Southern Cape (2006).

Molekwa’s (2013) recent research into the climatology and spatial distribution of cut-off lows in South Africa, focusing on the eastern and south-eastern parts of the country, highlights their significance and regularity as flood-producing systems. For the 31-year period from 1979 to 2009, 212 cut-off lows were identified. Sixty of the 138 cut-off lows over the Eastern Cape for this period were associated with heavy rainfall, mainly along the coastal regions. Regarding seasonality, March-April-May (MAM) and June-July-August (JJA) are the seasons with the highest frequencies of cut-off lows for the Eastern Cape. More than 40% of cut-off lows lasted for 3 to 4 days, with the majority of rain normally falling on one of the 4 days. For the 31-year period, Port Alfred averaged 616 mm of rainfall per annum, with approximately 33% of this total resulting from cut-off lows. These statistics (Molekwa 2013) are significant in the context of this study.

## Research methodology

### Approach

A combined quantitative and qualitative approach was adopted for the purposes of this study. This proved to be the most suitable approach as the study uses both quantitative and descriptive data to provide a comprehensive account of the disaster (Kumar 2014).

Mixed methods were used to analyse the meteorological information and data, whilst qualitative methods were used to critically review the functions and actions of the various role-players during the flood event (De Vos et al. 2011; Kumar 2014).

### Research setting

The research was set predominantly in the town of Port Alfred in the Eastern Cape Province, but included nearby coastal towns such as Kenton-on-Sea and Bushman’s River (Figure 1). Importantly, adjacent township areas were included in the study, as much of the damage occurred there. The research was carried out from January 2013 to December 2014. Participants in the study included the inhabitants of Port Alfred. The qualitative research was completed by the first author, whilst the quantitative research, based primarily on the climatic data, was completed by the second author.

### Data collection

Information regarding the actual flood event was obtained from the following secondary sources: local and regional newspaper reports as well as local and district municipal reports. The meteorological data and weather warning information were sourced from the SAWS. The daily weather bulletins for the relevant period were downloaded from the SAWS website. These daily weather bulletins have a surface synoptic chart showing the location of the weather systems on the day as well as a brief discussion of the weather systems, temperatures and areas of rainfall. The second author, who is a weather forecaster qualified in the field of meteorology, analysed these charts and wrote the synoptic overview given in the meteorological review. Daily rainfall data and wind speeds are recorded by the SAWS and were obtained from the Port Elizabeth Weather Office. Satellite imagery was downloaded from the online archives of the EUMETSAT (2014) Earth Observation Portal.

Primary data were obtained from semi-structured interviews with key role-players (Verhoeven 2011).

## Findings and discussion

### Meteorological review 15–23 October 2012

This section includes an analysis of the synoptic situation and rainfall on the days prior to and during the flooding in Port Alfred. Such an approach can shed more light on the soil conditions, as well as the possible height of the rivers and water table at the time of the flooding, which may have exacerbated the impact.

#### Synoptic overview

**15 October 2012:** A surface high-pressure system was located south-west of the country, bringing onshore (south-easterly) flow along the south-east coast. An upper air trough, lying west of the country, facilitated the development of showers and thundershowers and, together with the onshore flow, resulted in moderate to heavy rainfall.

**16 October 2012:** The surface high-pressure system moved eastwards, resulting in easterly to south-easterly flow into the Eastern Cape Province and, together with an upper air trough lying west of the country, resulted in showers and thundershowers.

**17 October 2012:** The surface high-pressure system lay to the south-east of the country, resulting in onshore flow into the Eastern Cape for the third consecutive day. The upper air trough made landfall on the west coast of the country and facilitated the development of widespread showers and thundershowers, resulting in heavy rain (≥ 50 mm) in places in the Eastern Cape.

**18 October 2012:** A surface low-pressure system developed just south of Durban, resulting in light south-easterly to south-westerly winds along the Eastern Cape coast.

**19 October 2012:** A cut-off low-pressure system developed over the south-western parts of the country with a surface low-pressure system along the south coast. Showers and thundershowers resulted across the Eastern Cape Province.

**FIGURE 3:**
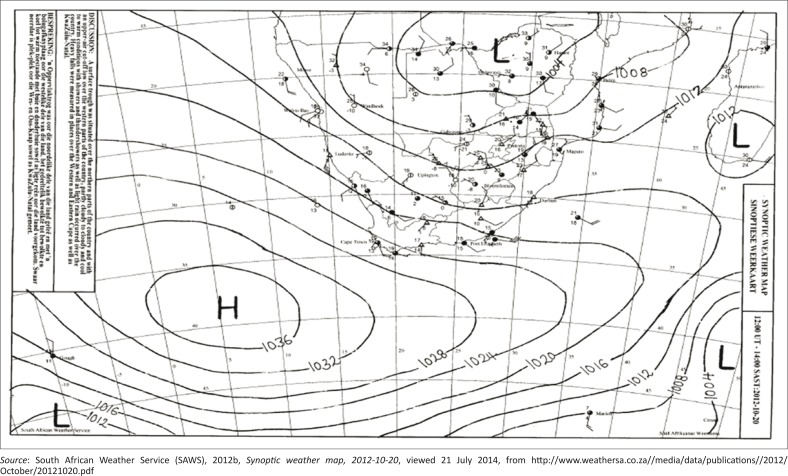
Surface synoptic chart on 20 October 2012 at 14:00 SAST.

**20 October 2012:** An upper air cut-off low was located over the western parts of the country with a surface high-pressure system extending a ridge along the south-east coast (Figure 3). This is known as a ‘black south-easter’, as strong south-easterly winds pushed moisture in from the Indian Ocean onto the south-eastern coastline.

**21 October 2012:** The upper air cut-off low remained over the south-western parts of the country; however, a surface low-pressure system replaced the high-pressure system of the previous day, cutting off the low-level moisture. Showers and thundershowers still occurred as a result of the effect of the cut-off low.

**22 October 2012:** The Atlantic Ocean surface high-pressure system extended a ridge along the south coast, bringing strong south-westerly winds along the coast with isolated showers and thundershowers in places.

**23 October 2012:** The Atlantic Ocean surface high-pressure system was located south of the country, ridging into the eastern parts of the province, bringing little rainfall to the western half of the Eastern Cape.

#### Rainfall and wind data analysis

Rainfall and wind data were retrieved from the SAWS for six stations from the area of Port Alfred and surrounds. Port Alfred Airport, Grahamstown and East London are automatic weather stations (AWS), which means that rainfall measurements are automated. However, Royal Port Alfred Golf Course, Alexandria and Kidd’s Beach are manual rainfall stations, where rain is measured by rain gauges and captured daily by individuals.

The rainfall data retrieved (Table 1) include two days prior to the cut-off low event, that is, 15 and 16 October 2012. Saturated soils prior to periods of heavy rainfall can greatly affect the occurrence of floods, hence these days were included.

**TABLE 1:** Daily rainfall values, in millimetres, recorded at rainfall stations in and around Port Alfred.

Date	Port Alfred Airport	Royal Port Alfred Golf Course	Alexandria	Grahamstown	Kidd’s Beach	East London	Total
15 Oct. 2012	53.8	6.0	31.3	38.5	4.5	20.2	154.3
16 Oct. 2012	6.6	142.0	9.8	42.0	9.2	13.4	223.0
17 Oct. 2012	165.4	3.0	83.5	89.5	21.6	55.0	418.0
18 Oct. 2012	2.4	53.0	7.3	67.0	1.3	1.4	132.4
19 Oct. 2012	23.2	0.0	23.4	75.0	13.8	14.2	149.6
20 Oct. 2012	129.4	116.0	82.0	98.5	25.1	30.6	481.6
21 Oct. 2012	2.0	4.0	9.7	0.0	0.1	0.2	16.0
22 Oct. 2012	2.4	1.5	23.0	0.0	0.6	0.0	27.5
23 Oct. 2012	0.8	0.0	1.1	0.0	2.3	10.0	14.2

**Total**	**386.0**	**325.5**	**271.1**	**410.5**	**78.5**	**145.0**	-

*Source*: Adapted from South African Weather Service (SAWS), 2012b, *Synoptic weather map, 2012-10-20*, viewed 21 July 2014, from http://www.weathersa.co.za//media/data/publications//2012/October/20121020.pdf

Moderate to heavy rainfall fell across the south-east coast and adjacent interior (including Grahamstown); 53.8 mm was recorded at Port Alfred Airport on 15 October. Conversely, only 6 mm of rain was recorded at the Royal Port Alfred Golf Course. These stations are approximately 2.7 km apart as the crow flies. This patchy distribution of rain is generally associated with convective rainfall, where thunderstorms producing the rainfall are classified as mesoscale phenomena of 3 km – 100 km wide (WMO 2008).

The SAWS classifies heavy rainfall as 50 mm or more. On 16 October, the Royal Port Alfred Golf Course recorded 142 mm of rain. This would have resulted in flooding in parts of Port Alfred. After these 2 days of rainfall, the soil would have been well saturated and disaster management role-players should have been on alert. If any more rainfall was expected in the next few days, flooding would most likely occur. On 17 October, Port Alfred Airport recorded 165.4 mm of rain. With the previous 2 days of rain this would have resulted in extensive flooding. On 20 October, 129.4 mm was recorded at Port Alfred Airport and 116 mm of rain at the Royal Port Alfred Golf Course.

Interestingly, heavy rainfall was recorded slightly inland at Grahamstown. For 6 consecutive days, the station recorded more than 35 mm of rain, with a maximum of 98.5 mm on 20 October. This amounted to a total of 410.5 mm in 6 days in an area upstream from Port Alfred. The Kowie River has its headwaters in the higher-lying Grahamstown area and drains south-eastward into Port Alfred (RSA & Department of Water Affairs and Forestry 2001). The town of Port Alfred is located on the Kowie River and therefore rainfall from upstream would have greatly contributed to the discharge volume and height of the river and water table at the time of the floods.

The heaviest rainfall was concentrated in the Grahamstown/Port Alfred region (Table 1). The heaviest rainfall occurred on 17 and 20 October 2012 across the entire south-east coast.

The average wind direction on the days with the heaviest rainfall at Port Alfred Airport, namely 15, 17 and 20 October, was 125 degrees with a compass direction of east-southeast (Table 2). Wind speeds averaged 2.7 m/s (9.7 km/h) and 6.5 m/s (23.4 km/h) on the 15th and 17th, respectively. The strongest wind speed on the days with significant rainfall recorded at the Port Alfred Airport was 6.5 m/s (23.4 km/h). This occurred on the same day as the heaviest rainfall at this station, indicating a direct relationship between the surface wind speed and the amount of rainfall recorded, given that the wind direction was ESE, which is an onshore flow for this area. Strong onshore flow would have accelerated low-level moisture from the warm Indian Ocean into this area to facilitate cloud development and rainfall.

**TABLE 2:** Average wind data for 15–23 October 2012 recorded at Port Alfred Airport.

Date	Wind direction (degrees)	Wind speed (m/s)
15 Oct. 2012	144	2.7
16 Oct. 2012	105	1.0
17 Oct. 2012	104	6.5
18 Oct. 2012	121	3.4
19 Oct. 2012	243	3.5
20 Oct. 2012	127	4.6
21 Oct. 2012	273	3.5
22 Oct. 2012	275	6.8
23 Oct. 2012	255	7.7

*Source*: Adapted from South African Weather Service (SAWS), 2012b, *Synoptic weather map, 2012-10-20*, viewed 21 July 2014, from http://www.weathersa.co.za//media/data/publications//2012/October/20121020.pdf

Singleton and Reason (2006) found that rainfall amounts were enhanced when onshore flow from the warm Agulhas Current collided with higher topography. This could account for the higher rainfall figures in Grahamstown which is situated inland at approximately 650 m above sea level.

#### Satellite imagery

**FIGURE 4:**
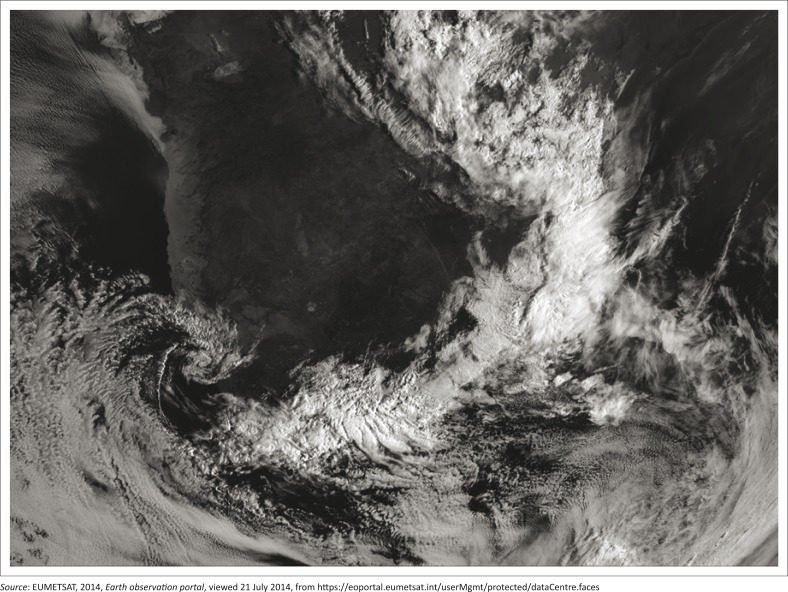
A high-resolution satellite image on 20 October 2012.

Figure 4 below shows convective cloud in the form of showers and thundershowers located on the south- east coast of the country as indicated by the very white colour, caused by the strong ridging high-pressure system over the sea and the upper air instability caused by the cut-off low. On the Western/Northern Cape coastline clockwise rotation in the cloud shows the location of the cut-off low. The cloud takes on a typical ‘comma’ shape associated with cut-off lows.

#### The role of disaster management, municipal officials and other agencies during the flood event

SAWS issued the severe weather watch (2–3 days’ lead time) and warning (24 hours’ lead time) of heavy rainfall and possible localised flooding for the south-eastern coast to disaster management by SMS well in advance of the event (SAWS 2012a). This warning was received by disaster management in both Ndlambe and Cacadu municipalities, yet there was a delay in communicating it to the relevant stakeholders and the general public (D. Shuping [Provincial Disaster Management Centre: Eastern Cape], pers. comm., 13 July 2014; K. van der Walt [National Sea Rescue Institute: Port Alfred], pers. comm., 13 July 2014). No action was taken by disaster management nor by the municipal officials until the flooding was already severe. Ndlambe Disaster Management issued a flood warning through the NSRI on 18 October that the river was expected to rise by 2 m, combined by a spring high tide (K. van der Walt [National Sea Rescue Institute: Port Alfred], pers. comm., 13 July 2014). However, this warning was already too late. Jetties were already breaking up and the marine walls of the marina were being undercut already by the Kowie River, which was surging at 14 knots.

This delay is seen as a direct result of a lack of any emergency or disaster management plan for the local municipality, which should focus on early warning, prevention and mitigation. In many cases, municipal managers and local officials are not too sure what action to take when weather warnings are issued (D. Shuping [Provincial Disaster Management Centre: Eastern Cape], pers. comm., 13 July 2014).

The disaster management official for Ndlambe was in office prior to and during the flood in Port Alfred, yet had no training or experience in managing disasters of such magnitude. As such, he did not take control of coordinating any relief or recovery activities. This responsibility was assumed by Cacadu Municipality, who had an official stationed in Port Alfred at the time of the flood.

A local joint operations centre (JOC) was set up at the fire station, but all role-players were not well represented and no person or organisation took charge of the overall coordination of relief activities (D. Shuping [Provincial Disaster Management Centre: Eastern Cape], pers. comm., 13 July 2014; K. van der Walt [National Sea Rescue Institute: Port Alfred], pers. comm., 13 July 2014). In addition, local residents who volunteered their assistance were turned away. The NSRI, who was a key role-player in rescue and relief operations during the flood (Lang 2012), was not invited to become a member of the JOC. This lack of coordination led to important role-players and organisations taking charge of their own areas of operation, but without the benefit of working together with other actors and agencies during the flood. This, in turn, resulted in a fragmented and poorly executed relief operation.

The accurate deployment of scarce resources and manpower to the worst affected areas was also affected by the poor management of the event. In a number of cases, local residents and communities teamed up to repair or temporarily rebuild structures such as low-lying causeways which had been washed away (Butler, Houzet & Williams 2012; Lang 2012).

In particular, the dilapidated and fragile storm water systems could not cope with the excessive amounts of run-off, which resulted in many homes and businesses being flooded or completely destroyed. Hardest hit areas in the Port Alfred area included South Downs, Medolino Caravan Park and New Rest and Nemato townships. Many residents had to be evacuated to higher-lying areas, where they were accommodated in temporary shelters and were supplied with food, blankets and mattresses. Tiger Brands Johannesburg donated some of the relief food supplies (Smith 2012).

Importantly, the entire storm water system for Port Alfred needed replacing. Application was made to national government for disaster relief funding of R29 million for the district, following the severe floods of 2010 and 2011 (Smith 2012). No funding was received; hence no infrastructural improvements could be brought about in Port Alfred and the surrounding communities.

A weakness in the entire operation was the lack of cooperation by disaster management with the local Department of Social Development, who were directly responsible for food and shelter assistance to victims during the flood. In fact, food parcels were delivered to needy families in the surrounding location more than three months after the flood (D. Shuping [Provincial Disaster Management Centre: Eastern Cape], pers. comm., 13 July 2014).

Ndlambe and Cacadu municipalities were both declared disaster districts after the flood and application was made to national government for flood relief, after damage surveys were conducted by local and provincial officials. A provincial state of disaster was declared in November 2012 (Nelson Mandela Bay Municipality 2013). Conservative estimates for infrastructural (excluding domestic) repairs were R120 million for Ndlambe Municipality alone (Cacadu District Municipality 2012).

## Conclusion and recommendations

The Port Alfred floods of 2012 were not exceptional, neither were they a climatological anomaly (Nott 2006). Research has shown that Port Alfred and the broader Eastern Cape coastline and adjacent interior are susceptible to flooding from cut-off lows at frequent and regular intervals. In a sense, the floods and the ensuing damage were predictable, given past events in the area.

The meteorological conditions which triggered the disaster have been thoroughly documented and analysed in this study. Cut-off lows are well understood climatological phenomena and their development and attributes have been accurately documented in the literature. In the floods of 2012, the impact was exacerbated by 2 days of heavy rain prior to the actual flooding caused by the cut-off low weather system. Water tables and river levels were already high, which led to increased volumes of storm water run-off and resultant damage to infrastructure and private property. At this stage, disaster management should have been put on high alert, but no concrete action was taken.

The poor state of the existing infrastructure, in particular the storm water systems, also exacerbated the impact of the flood. Low-lying areas in the local informal settlements and township, as well as the properties in the marina development, were particularly hard hit as a result. Weather impacts were exacerbated by the adverse social conditions in the highly vulnerable township areas.

SAWS gave relevant stakeholders in Ndlambe and Port Alfred sufficiently early warning, but disaster management and other municipal officials’ failure to take decisive and appropriate action delayed prevention, mitigation and relief operations. The lack of proper coordination by disaster management in the area certainly exacerbated the situation. In addition, the lack of an emergency or disaster management plan for Ndlambe Municipality and Port Alfred is a serious shortcoming that militates against any preventative measures which should have been in place. Limited capacity and poorly trained disaster management officials in the area compounded the situation further.

In light of the aforementioned, the following recommendations are made in order to improve proactive disaster risk reduction for Port Alfred and the broader Ndlambe Municipality:

- the urgent development of a local disaster management plan for Ndlambe Municipality
- the establishment of a local disaster management advisory forum for Ndlambe Municipality
- the training of local disaster management officials and local councillors in proactive disaster risk reduction approaches
- the training of local disaster management officials and councillors to better understand severe weather warnings issued by SAWS, and how to disseminate such information to relevant stakeholders and role-players
- the allocation of better resources to deal with future events of this nature
- improved collaboration and building of sound working relationships between disaster management and the local departments of Social Development and Human Settlements.
